# Hypoxia Limits Inhibitory Effects of Zn^2+^ on Spreading Depolarizations 

**DOI:** 10.1371/journal.pone.0075739

**Published:** 2013-11-22

**Authors:** Isamu Aiba, C. William Shuttleworth

**Affiliations:** Department of Neurosciences, University of New Mexico, Albuquerque, New Mexico, United States of America; Albany Medical College, United States of America

## Abstract

Spreading depolarizations (SDs) are coordinated depolarizations of brain tissue that have been well-characterized in animal models and more recently implicated in the progression of stroke injury. We previously showed that extracellular Zn^2+^ accumulation can inhibit the propagation of SD events. In that prior work, Zn^2+^ was tested in normoxic conditions, where SD was generated by localized KCl pulses in oxygenated tissue. The current study examined the extent to which Zn^2+^ effects are modified by hypoxia, to assess potential implications for stroke studies. The present studies examined SD generated in brain slices acutely prepared from mice, and recordings were made from the hippocampal CA1 region. SDs were generated by either local potassium injection (K-SD), exposure to the Na^+^/K^+^-ATPase inhibitor ouabain (ouabain-SD) or superfusion with modified ACSF with reduced oxygen and glucose concentrations (oxygen glucose deprivation: OGD-SD). Extracellular Zn^2+^ exposures (100 µM ZnCl_2_) effectively decreased SD propagation rates and significantly increased the initiation threshold for K-SD generated in oxygenated ACSF (95% O_2_). In contrast, ZnCl_2_ did not inhibit propagation of OGD-SD or ouabain-SD generated in hypoxic conditions. Zn^2+^ sensitivity in 0% O_2_ was restored by exposure to the protein oxidizer DTNB, suggesting that redox modulation may contribute to resistance to Zn^2+^ in hypoxic conditions. DTNB pretreatment also significantly potentiated the inhibitory effects of competitive (D-AP5) or allosteric (Ro25-6981) NMDA receptor antagonists on OGD-SD. Finally, Zn^2+^ inhibition of isolated NMDAR currents was potentiated by DTNB. Together, these results suggest that hypoxia-induced redox modulation can influence the sensitivity of SD to Zn^2+^ as well as to other NMDAR antagonists. Such a mechanism may limit inhibitory effects of endogenous Zn^2+^ accumulation in hypoxic regions close to ischemic infarcts.

## Introduction

Spreading depolarization (SD) is a slowly propagating, feed-forward event that initiates from coordinated depolarization of a volume of tissue. Local elevations of extracellular potassium and/or glutamate then appear to drive near complete depolarization of surrounding tissue. Mechanisms of SD have been extensively studied in animal models (reviewed in [1]), and recent clinical studies strongly suggest that SD can be frequent in the context of acute brain injury [2,3]. SD and related events (anoxic depolarization, peri-infarct depolarizations) appear to occur spontaneously in regions that become involved in the infarct core, as well as in surrounding tissues. The cumulative metabolic burden of repetitive SDs that occur in the hours and days following injury appears to increase the volume of tissue involved in an infarct, and there is therefore considerable interest in finding effective approaches to limit the incidence of SDs [4,5].

Zn^2+^ is highly concentrated in synaptic vesicles of many glutamatergic neurons and can be released into the extracellular space during SD [6]. We recently showed that extracellular Zn^2+^ accumulation can limit SDs generated in normoxic conditions *in vivo* and *in vitro* [7]. Extracellular Zn^2+^ can antagonize NMDARs [8], and such a mechanism could be one explanation for decreased SD incidence. In contrast to the potentially protective effects of extracellular Zn^2+^, excessive intracellular Zn^2+^ accumulation contributes to neuronal injury. Transmembrane flux of Zn^2+^ can occur via a range of voltage-dependent cation channels and selective Zn^2+^ transporters [9-11]. A number of influential studies have demonstrated toxic roles for intracellular Zn^2+^ accumulation in ischemic brain injury [10,12,13], and with regards to SD, it is noted that intracellular Zn^2+^ accumulation can contribute to initiation of some forms of SD [14], possibly by providing an additional metabolic challenge to tissues [15]. Thus the net effects of Zn^2+^ on stroke progression are likely a balance between these extracellular and intracellular actions. The factors that influence this balance are not well described, and may be important for development of effective therapeutic interventions based on Zn^2+^.

In the present study, we investigated whether inhibitory effects of Zn^2+^ on SD were influenced by oxygen or glucose availability. The results show a dependence of Zn^2+^ inhibition on oxygen concentration, which could be contributed to by redox modulation. Such a mechanism may provide an additional link between tissue metabolism and the pharmacological sensitivity of SD in ischemic conditions.

## Experimental Procedures

### 1. Ethics Statement

All experimental procedures were carried out in accordance with the recommendations in the Guide for the Care and Use of Laboratory Animals of the National Institutes of Health, the Animal Welfare Act and US federal law. The experimental procedures were approved by the Institutional Animal Care and Use Committee (IACUC) at the University of New Mexico. 

### 2. Animals and slice preparation

Brain slices were prepared from 4-10 week old mice of either sex, from C57Bl/6 or FVB/N strains. The choice of strains was based on pervious work, as we previously characterized Zn^2+^ sensitivity of SD in FVB/N mice and then included mice of the C57BL/6 strain to allow comparison with ZnT3 KO animals [7]. Since some parts of the present study were conducted in parallel with that prior work, both strains are included in this report. Importantly, throughout the present study, pharmacological intervention was tested by interleaving vehicle and test slices obtained from the same experimental animals to control for any potential animal variability. Mice strains and sexes are indicated in each Figure legend. 

Brain slices were prepared as previously described [7]. Briefly, mice were deeply anesthetized with a ketamine/xylazine mixture and decapitated. Brains were then extracted into ice cold cutting solution (mM: 220 Sucrose, 6 MgSO_4_, 3 KCl, 1.25 Na_2_HPO_4_, 25 NaHCO_3_, 10 glucose, 0.2 CaCl_2_, equilibrated with 95% O_2_ / 5% CO_2_) and sliced with a vibratome at 350 µm. Slices were then allowed to recover in ACSF (mM: 126 NaCl, 1 MgSO_4_, 3 KCl, 1.25 Na_2_HPO_4_, 25 NaHCO_3_, 10 glucose, 2 CaCl_2,_ equilibrated with 95% O_2_/5% CO_2_) at 35°C for 1 hour, and then transferred to room temperature ACSF and held until transfer to the recording chamber. Recordings were made in the submerged configuration (RC-27 chamber, Warner Instruments, Hamden, CT), continuously superfused with ACSF at 2 ml/min at 32°C.

### 3. SD recordings and stimuli

**Figure 1:**
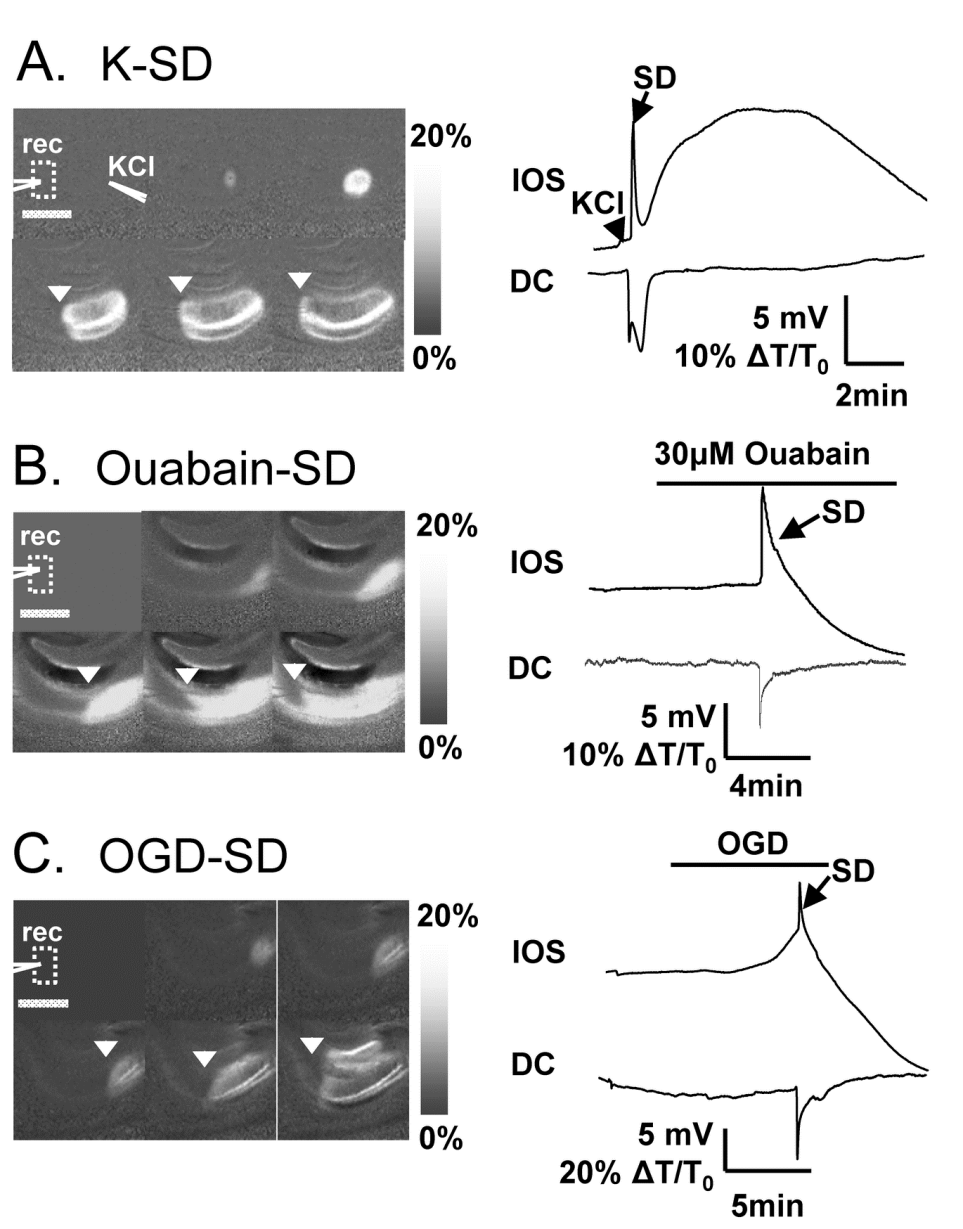
Representative SD responses generated by the three different stimuli. **A**: SD generated by KCl microinjection. Left panels show a series of 6 images (2 second intervals) of intrinsic optical signals (ΔT/T_0_) following KCl microinjection ejected via a micropipette (location indicated by “KCl”). Intensity increases in these ratio images are indicated by brightening of the image, and the advancing wavefront of SD is marked by the arrowheads. Traces at right show IOS and electrical signals recorded from the same preparation. IOS signals were recorded from a region of interest indicated by the box, and extracellular potential changes were recorded with a microelectrode placed at position “rec”. Scale bar: 400 µm. **B**: SD generated by exposure to the Na^+^/K^+^/ATPase inhibitor ouabain (30 µM). Details of the figure are as described for A, with the DC shift and propagating wavefront of SD being generated ~5 min after the onset of ouabain exposure. **C**: SD generated by exposure of slice to OGD. Similar to ouabain-SD, OGD-SD was initiated after a significantly delay, and rates of SD propagation could be calculated by advancing wavefront of IOS signals. Data were obtained from slices obtained from male C57BL/6 mice.

SD was detected as a sharp DC potential change recorded with an extracellular recording electrode, accompanied by a spreading wave of intrinsic optical signal (IOS) increases (Figure **1**). Low resistance glass electrodes (0.5-1 MΩ, filled with ACSF) were placed in the CA1 dendritic subfield. Signals were amplified with a Multiclamp 700A amplifier, digitized (Digidata 1322A) and analyzed with pClamp9.2 software (Molecular Devices, CA). IOS (>575nm) were acquired at 0.5-1 Hz using a CCD camera controlled by Till Vision software (Version 4.0). SD propagation rates were analyzed after SDs were fully established (>8 s after initial observation), and rates were averaged from at least 3 consecutive images. 

SD was generated by one of three stimuli: 1) K-SD was induced by local ejection of 1 M KCl via a glass micropipette (~1 µm tip diameter, 40 psi). The ejection volume from a 10 ms pulse was estimated to be ~10 nl, based on the comparison between the diameter of droplet from 10-20 ejection and a reference volume [16]. The KCl ejection pipette was placed in the hippocampal CA1 dendritic subfield >300 µm distant from the recording site and 50 µm below the slice surface. The threshold for K-SD was determined by escalating the duration of KCl microinjections, began with a 10 ms pulse, followed by doubling of the stimulus duration every 5 minutes until a propagating event was generated. Ouabain-SD was generated by superfusion with ACSF containing 30 µM ouabain, equilibrated with varying concentrations of O_2_). Oxygen and glucose deprivation (OGD)-SD was generated by superfusion with a modified ACSF, prepared by complete substitution of O_2_ with N_2_, and 85% substitution of glucose with sucrose (i.e. 1.5 mM glucose, 8.5 mM sucrose). 

### 4. Drugs

D-AP5, MK801 and Ro25-6981 were obtained from Sigma. D-AP5 was dissolved in water at a stock concentration of 10 mM. MK801 and Ro25-6981 were dissolved in DMSO at 100 mM and 10 mM, respectively. Matched final DMSO concentrations were used in vehicle control experiments. In the experiments that included ZnCl_2_, 200 µM histidine was always included in the ACSF. This method can effectively limit Zn^2+^ precipitation in phosphate containing solutions [17]. Histidine supplementation was preferred for the present studies over phosphate removal, to avoid potential negative effects on metabolism. As reported previously [7], no confounding effects of histidine alone were detected on SD characteristics.

### 5. NMDAR current recording

Whole-cell NMDAR currents were recorded from single CA1 pyramidal neurons. Whole-cell clamp was obtained with patch pipettes (2-3 MΩ) with internal solution containing (in mM): 130 cesium-methanesulfonate, 10 Hepes, 0.5 CaCl_2_, 8 NaCl, 10 EGTA, 5 QX314, 2 Na_2_ATP, 0.3 Na_3_GTP, pH adjusted to 7.2. Neurons were dialyzed for at least 10 minutes prior to recordings. Excitatory postsynaptic NMDAR currents (EPSC_NMDA_) were evoked by iontophoretic delivery of glutamate from high resistance electrodes (20-30 MΩ, filled with 1 M sodium glutamate). Glutamate electrodes were placed <30 µm from the putative locations of apical dendritic trees of whole-cell patched neurons. Recordings were made in the presence of 20 µM DNQX and 15 µM bicuculline, and evoked glutamate currents were initiated at +30 mV. The duration and intensity of glutamate stimulation was adjusted for each recording in order to obtain evoked currents less than 1 nA in amplitude and 1 s duration (typical stimuli: 10-30 µA, 0.5-3 ms). Control experiments utilizing the same parameters with 1 M NaCl-filled stimulation electrodes did not evoke postsynaptic currents (n=4). Input resistance and series resistance was estimated ~500 ms prior to testing NMDA currents. Recordings in which series resistance changed by >20% were excluded from the analysis. 

### 6. Statistics

Student *t*-tests or ANOVA with post hoc Turkey analysis were used for statistical analysis unless otherwise described. P<0.05 was considered to be statistically significant.

## Results

### 1. Sensitivities of K-SD, ouabain-SD and OGD-SD to ZnCl_2_

**Figure 2:**
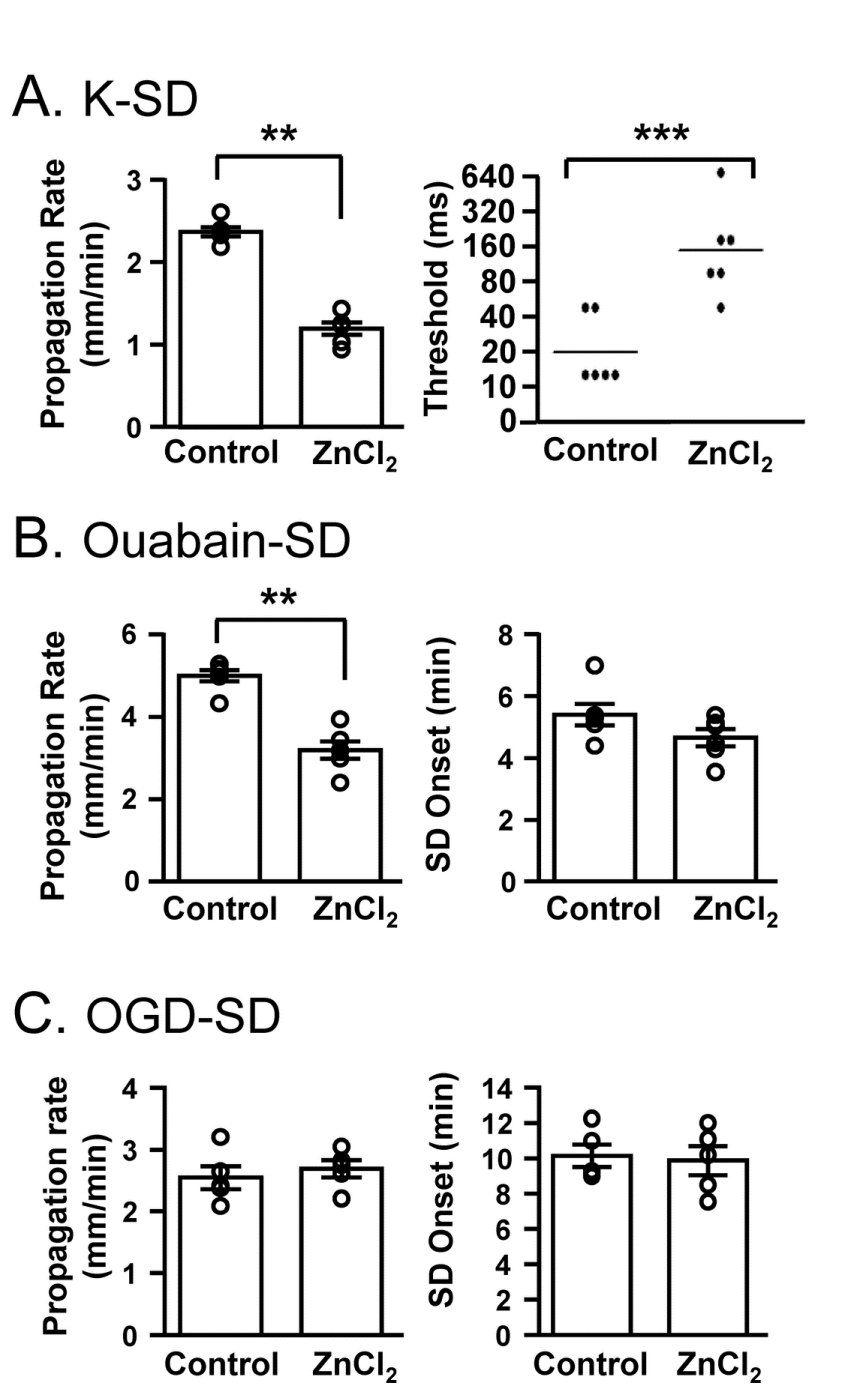
Differential sensitivities of K-SD, ouabain-SD and OGD-SD to ZnCl_2_. Slices were pre-exposed to ZnCl_2_ (100 µM, 10 min) before application of one of the three SD stimuli. **A**: Propagation rates of K-SD were significantly reduced, and the threshold for SD onset (see Methods) was significantly increased in the same preparations (n=6, **p<0.01, ***p<0.005). **B**: ZnCl_2_ significantly reduced propagation rates of ouabain-SD, with no significant decrease in the time to SD onset (n=5, **p<0.01). **C**: In contrast to the two other stimuli, ZnCl_2_ exposures were without effect on the propagation rates of OGD-SD (n=5). Data were obtained from slices prepared from male FVB/N mice.

Figure **1** shows the general features of SD generated by the three stimuli used in the study: A) KCl microinjection, B) exposure of slices to the Na^+^/K^+^-ATPase inhibitor ouabain, or C) superfusion with modified ACSF with reduced oxygen and glucose (OGD, see Methods). The sensitivity of these three types of SD to extracellular Zn^2+^ was examined by bath application of 100 µM ZnCl_2_ (Figure **2**), and experimental slices were interleaved with control slices throughout. As reported previously [7], ZnCl_2_ reliably inhibited the rate of propagation of K-SD (Figure 2A), and this was attributed to extracellular actions of Zn^2+^ in this model [7]. In addition, ZnCl_2_ significantly increased the threshold for K-SD initiation. This new observation of increased threshold is consistent with inhibition of SD frequency seen in an *in vivo* CSD model [7]. 

We next examined sensitivity of ouabain-SD (Figure 2B) to ZnCl_2_ in 95% O_2_. The concentration of ouabain (30 µM) was previously demonstrated to have significant sensitivity to NMDAR antagonists [14]. The propagation of ouabain-SD was significantly inhibited by ZnCl_2_ (Figure **2B**), while SD initiation (as evaluated from SD onset time) was unaffected.

Figure 2C shows the lack of effect of ZnCl_2_ on OGD-SD. In the present study, the OGD solution lacked any added O_2_ and glucose was reduced to 1.5 mM, as these conditions proved effective for testing the pharmacosensitivity of OGD-SD (see below). Exposure to OGD resulted in an initial generalized IOS signal increase, and SD was then optically detected as a propagating band of sharply enhanced IOS. ZnCl_2_ was without effect on propagation rate or onset of OGD-SD (Figure **2C**). Because of the limited solubility of ZnCl_2_ in ACSF (see Methods), we could not test higher ZnCl_2_ concentrations to determine whether resistance to ZnCl_2_ was absolute. However these results revealed a large difference in the ZnCl_2_ sensitivity of OGD-SD, compared with SD generated under conditions of abundant oxygen (K-SD and ouabain SD). 

### 2. Oxygen concentration strongly influenced the *Zn* ^*2+*^ sensitivity of SD propagation

**Figure 3:**
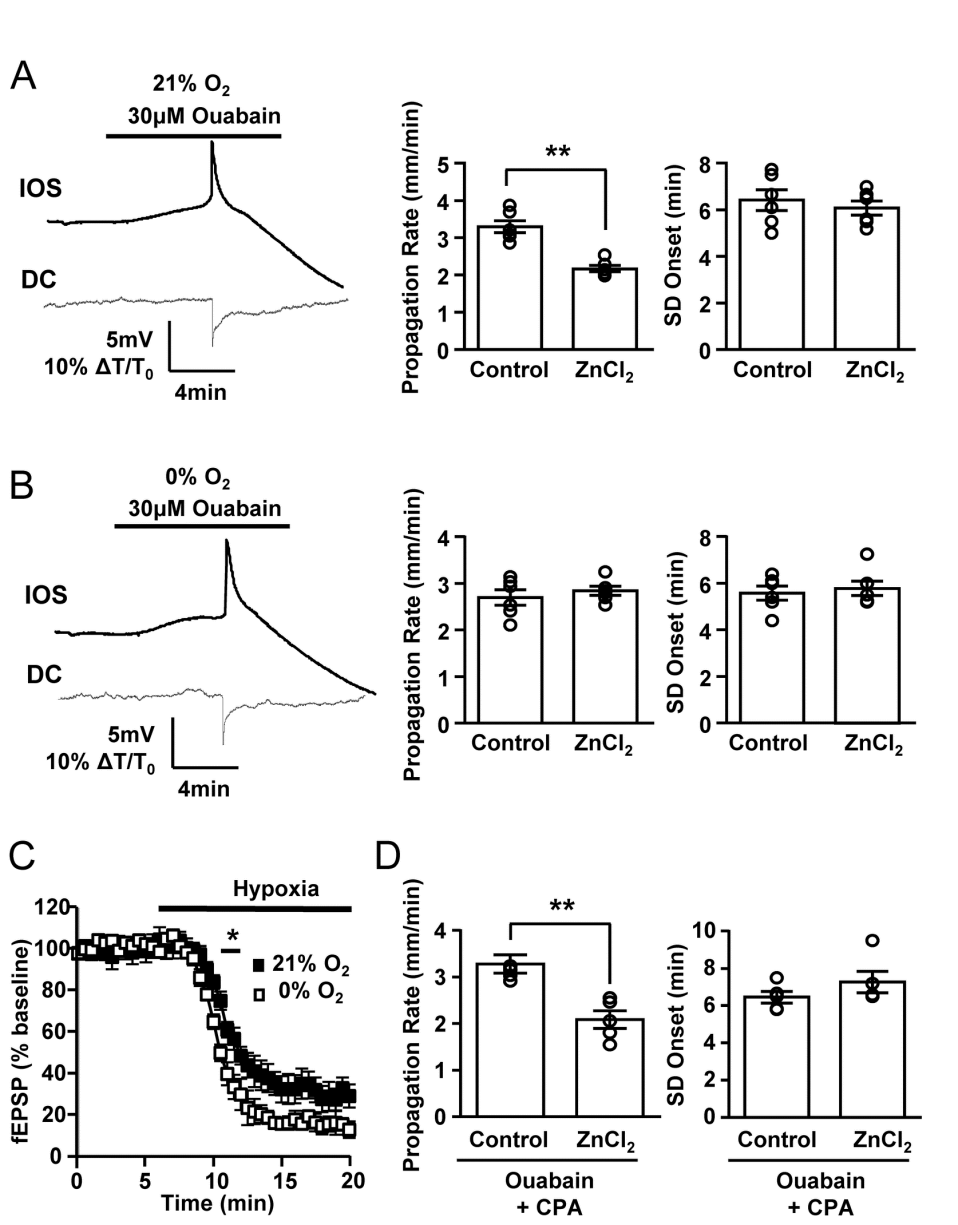
Changing O_2_ availability dictates sensitivity to ZnCl_2_. **A**&**B**: Left panels show representative traces of ouabain-SD generated under different oxygen concentrations and right panels show summary effects on propagation rate and SD onset. ZnCl_2_ (100 µM) application produced a significant decrease in SD propagation rate in 21% O_2_, but inhibitory effects of Zn^2+^ were not observed in in 0% O_2_ (n=6 each, **p<0.01). **C**. Reduction of field excitatory postsynaptic potentials (fEPSP) during hypoxia. Baseline recordings were in 95% O_2_, before exchange with either 21% or 0% O_2_ (replaced with N_2_, see Methods). * p<0.05, n=5 each. **D**. Suppression of synaptic transmission with the A1 receptor agonist CPA (300 nM, in 95% O_2_) decreased SD propagation rate, but did not prevent inhibition by ZnCl_2_ (n=5, **p<0.01) Experiments were performed on slices obtained from male FVB/N mice.

We next examined whether the selective removal of oxygen and/or glucose could be sufficient to render SD insensitive to ZnCl_2_. Ouabain was a suitable SD stimulus to test these possibilities, because in the absence of oxygen/glucose, ouabain exposure still reliably generates SD, whereas K-SD is difficult to generate. Figures 3A&B show that the ZnCl_2_ sensitivities of ouabain SD generated with these lower O_2_ concentrations were quite different. Thus SD propagation in 21% O_2_ was significantly inhibited by ZnCl_2_ (Figure **3A**, similar to the 95% O_2_ condition). In contrast, no inhibitory effect of ZnCl_2_ on the propagation of ouabain-SD was observed in 0% O_2_ (Figure **3B**). 

Hypoxia inhibits synaptic transmission via adenosine accumulation and A_1_ receptor activation [18]. We therefore examined whether increased suppression of synaptic transmission could contribute to greater resistance of responses to ZnCl_2_ in 0% O_2_. Excitatory postsynaptic potentials (fEPSP) were evoked by using a bipolar stimulating electrode placed in stratum radiatum (70μs, 0.1Hz). Figure 3C shows that exposure to either 21% O_2_ or 0% O_2_ ACSF effectively reduced fEPSP amplitude, although suppression was somewhat stronger in under 0% O_2_ than in 21% O_2_. We therefore tested whether fully blocking synaptic activity with the potent A_1_ receptor agonist, N^6^-cyclopentyladenosine (hereafter referred to as CPA, 300 nM) would mimic effects of hypoxia on ZnCl_2_ sensitivity. Exposure to CPA in 95% O_2_ abolished evoked potentials (see also 19), and decreased propagation rate of ouabain-SD to a similar level as observed in 21% and 0% O_2_. However CPA did not prevent inhibition of SD by ZnCl_2_ (Fig, 3D), indicating that changes in basal synaptic strength is unlikely to explain the oxygen-dependent differences in the sensitivity of ouabain-SD to Zn^2+^.

We next examined the influence of glucose deprivation in the ouabain-SD model. SD was reliably generated by ouabain solutions that lacked all added glucose (in ACSF 95% O_2_ and glucose substitution with sucrose). Under these conditions, SD propagation almost identical to control experiments (Figure 2C), and sensitivity to ZnCl_2_ was maintained (SD propagation rates, control: 4.50 ± 0.44 mm/min, ZnCl_2_: 2.71 ± 0.13 mm/min, n=5, *p*<0.01). These results argue against a potential role of glucose availability in the lack of ZnCl_2_ sensitivity of OGD-SD. 

### 3. Resistance of OGD-SD to ZnCl_2_ reversed by the protein oxidizers

**Figure 4:**
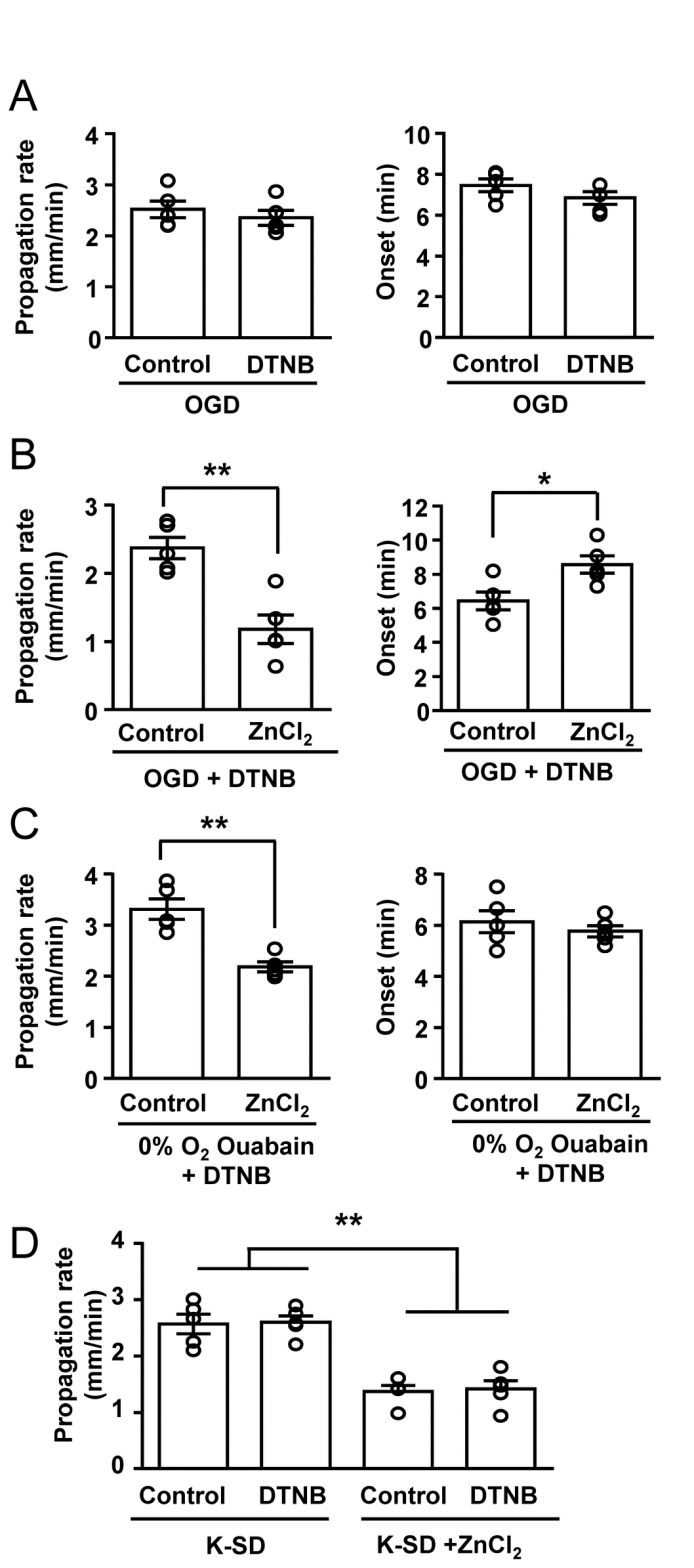
The protein oxidizer DTNB was sufficient to confer ZnCl_2_ sensitivity in anoxic conditions. **A**: DTNB pre-exposure (0.5 mM, 10 min) by itself had no effect on either OGD-SD propagation rate (left) or onset (right), n=5. **B**: In the presence of DNTB, ZnCl_2_ (100 µM) effectively inhibited OGD-SD propagation rate, and delayed OGD-SD onset (n=5, *p<0.05, **p<0.01). **C**: Similarly, ouabain-SD generated in 0% O_2_ was inhibited by ZnCl_2_ in DTNB pre-treated slices. **D**: Under conditions where ZnCl_2_ was already effective, DTNB did not further enhance ZnCl_2_ sensitivity. Illustrated here is a lack of effect of DTNB alone on K-SD, and lack of effect on the inhibition of K-SD by ZnCl_2_ (n=5, **p<0.01). These experiments were performed in slices obtained from C57BL/6 (4 female and 6 male mice).

The results above suggested that O_2_ concentration can be sufficient to affect the sensitivity of SD to extracellular ZnCl_2_. One potential explanation for these results is that severe hypoxia modulates Zn^2+^ sensitivities of target proteins by causing a reducing shift in extracellular redox potential. This possibility was tested by pre-exposure to the protein oxidizer 5,5'-dithiobis-(2-nitrobenzoic acid) (DTNB). DTNB has a poor membrane permeability [20] and has been used to modify redox modulation of many proteins, including extracellular domains of NMDARs [21,22]. A previous study has shown that a high concentration of DTNB (2 mM) significantly inhibited SD triggered by hypoxia [23], and consistent with this, 2 mM DTNB blocked OGD-SD in our recording conditions (n=3). A lower concentration of DTNB (0.5 mM) was found more appropriate for the current studies, since it did not prevent SD initiation, but likely retains an ability to prevent anoxia-induced redox modulation [24,25]. DTNB treatment (0.5 mM, 10 min, 95% O_2_) alone had no effect on OGD-SD, as assessed by onset time and propagation rate (Figure **4A**). However DTNB pre-treatment significantly increased the sensitivity of OGD-SD to ZnCl_2_. Thus ZnCl_2_ significantly decreased propagation rates and also delayed the onsets of OGD-SD in DTNB preexposed slices (Figure **4B**). Similarly, propagation of ouabain SD generated in 0% O_2_ (which was previously shown to be insensitive to ZnCl_2_; see Figure 3B), became sensitive to ZnCl_2_ in DTNB pre-exposed slices (Figure **4C**). A similar effect was seen with another oxidant oxidized glutathione (GSSG: 0.5mM, 10 min pre-exposure; propagation rates, control with GSSG: 2.26 ± 0.11 mm/min, ZnCl_2_ with GSSG: 1.55 ± 0.10 mm/min, n=5, *p*<0.01), supporting a role of redox modulation in the Zn^2+^ sensitivity of OGD-SD.

DTNB alone was without effect on the K-SD propagation rate and threshold. In addition, the ZnCl_2_ sensitivity of K-SD was unaffected (propagation rate Figure **4D**, threshold *p*>0.50, n=5). Taken together, these results suggest that extracellular redox modulation under severe hypoxia can be, at least in part, responsible for the lack of Zn^2+^ sensitivity of SD.

As an additional control, we examined whether intracellular Zn^2+^ accumulation could contribute to inhibitory effects of ZnCl_2_ exposures, specifically in the conditions where a ZnCl_2_ effect was revealed by DTNB. We examined this possibility by loading Zn^2+^ intracellularly by exposure to a Zn^2+^-ionophore complex (ZnPyr: 100 µM ZnCl_2_ and 1 µM pyrithione) for 10 minutes. In order to exclude the effects of residual extracellular ZnCl_2_, slices were then briefly washed with ACSF (3 minutes) before SD was generated in nominally Zn^2+^-free OGD solutions. Intracellular Zn^2+^ loading did not affect the propagation rate or onset time of OGD-SD in DTNB (propagation rates, control: 2.42 ± 0.15 mm/min, ZnPyr: 2.60 ± 0.24 mm/min; SD onset, control: 7.24 ± 0.39 min, ZnPyr: 7.71 ± 0.44 min, n=5, *p*>0.5). These results argue against a possibility of intracellular Zn^2+^ accumulation contributing to ZnCl_2_ inhibition of SD, and support the idea that Zn^2+^ is unable to inhibit OGD-SD because of anoxic redox modulation of extracellular sites.

### 4. Hypoxic modulation of NMDARs

NMDA receptors are one well-described target of Zn^2+^ [26], and Zn^2+^ sensitivity of NMDAR has been shown to be redox sensitive [27]. In addition, hypoxia modulates sensitivity to synthetic antagonists [28]. We thus tested whether the ZnCl_2_ sensitivity of NMDAR could also be decreased by hypoxia-dependent redox modulation. NMDAR dependent whole-cell currents were evoked by localized glutamate iontophoresis at +30 mV in the presence of GABA_A_ and AMPA receptor antagonists (see Methods). Under these conditions, evoked whole-cell currents were almost completely blocked by D-AP5 (10 µM AP5, 95.7 ± 5.0% inhibition, n=3), verifying them as isolated NMDAR currents. In contrast to synaptically-evoked fEPSP responses (see Figure 3A above), glutamate iontophoresis reliably evoked significant NMDAR currents in both 21% and 0% O_2_. 

**Figure 5:**
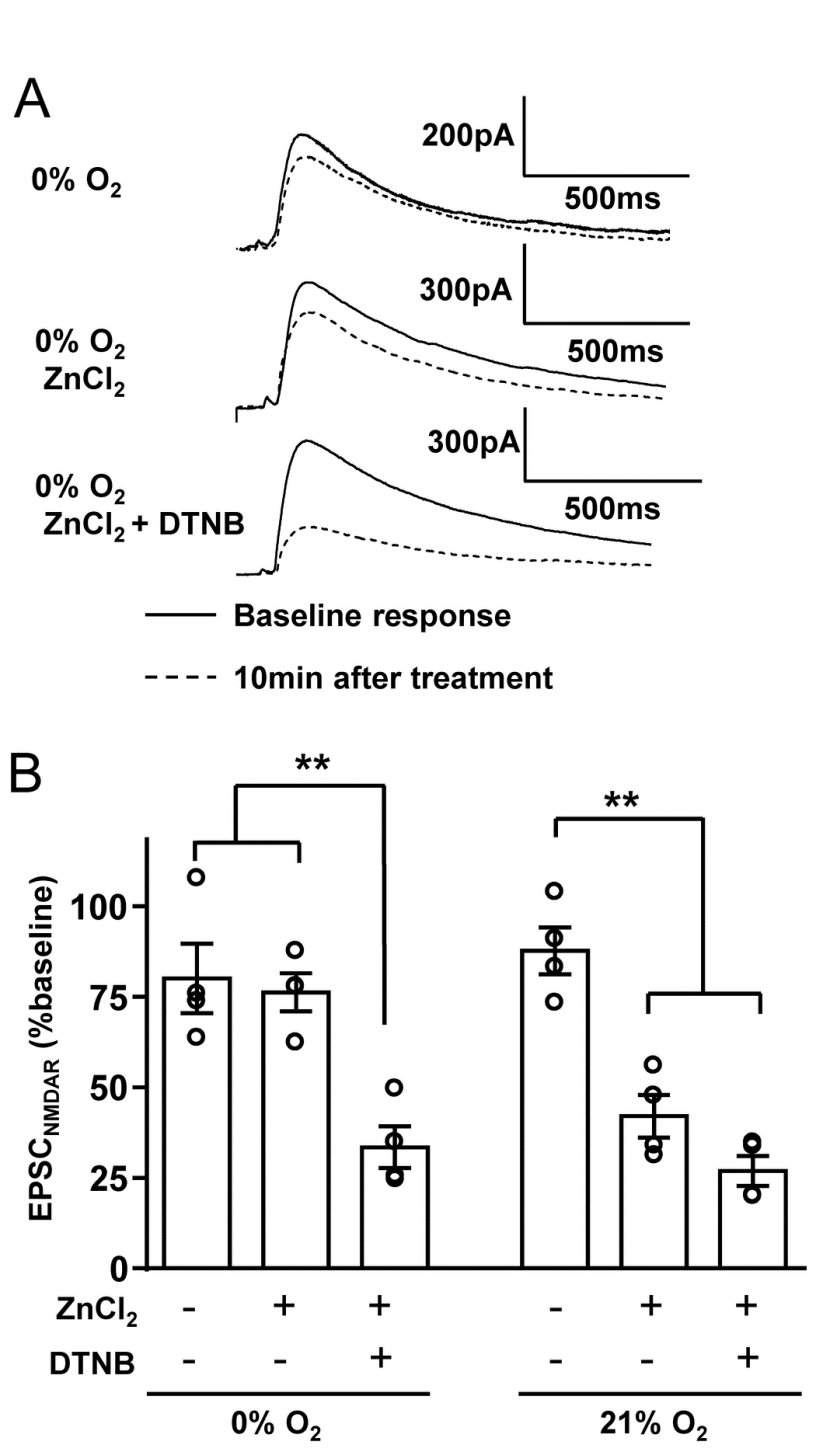
Zn^2+^ inhibition of NMDAR current was sensitive to hypoxic redox modulation. **A**: Whole-cell NMDAR currents were evoked by localized glutamate iontophoresis (see Methods). The effects of hypoxia and ZnCl_2_ were evaluated by comparing the amplitudes of baseline responses (average of 9 traces in 95% O_2_, solid lines in each panel) with the average of 4-6 responses obtained after 10-15 min exposure to either hypoxic solutions, or hypoxic solutions containing ZnCl_2_ (dashed lines in each panel). The top pair of traces shows baseline responses in 95% O_2_ (solid trace) and a small decrease in the same neuron obtained after 10 min exposure to 0% O_2_ (dashed trace). The middle pair of traces show that ZnCl_2_ exposure caused little additional run-down of evoked NMDAR current, when tested in 0% O_2_. However when slices were exposed to DTNB in 0% O_2_, ZnCl_2_ caused a large decrease in current amplitude. **B**: Summary data from sets of data illustrated in A (left), and also similar experiments conducted in 21%O_2_ ACSF (right) n=4 for each condition, **p<0.01). Slices obtained from C57BL/6 (1 female and 6 male mice) were used for these analyses.

The effects of ZnCl_2_ exposures on NMDAR currents were compared with time-control experiments. In 95% O_2_, ZnCl_2_ significantly inhibited NMDAR currents (100 µM ZnCl_2_, 79.7 ± 4.1% inhibition, n=3). In 0% O_2_, addition of ZnCl_2_ alone was almost without effect on NMDAR currents, however sensitivity was revealed by pretreatment with DTNB (Figure **5A&B**). In 21% O_2_, 100 µM ZnCl_2_ significantly decreased NMDAR currents and similar inhibition was observed when tested in DTNB pre-exposed slices (Figure 5A&B). These results suggest that similar to SD, the Zn^2+^ sensitivity of NMDAR may be attenuated by a mechanism involving hypoxia-dependent redox modulation. 

**Figure 6:**
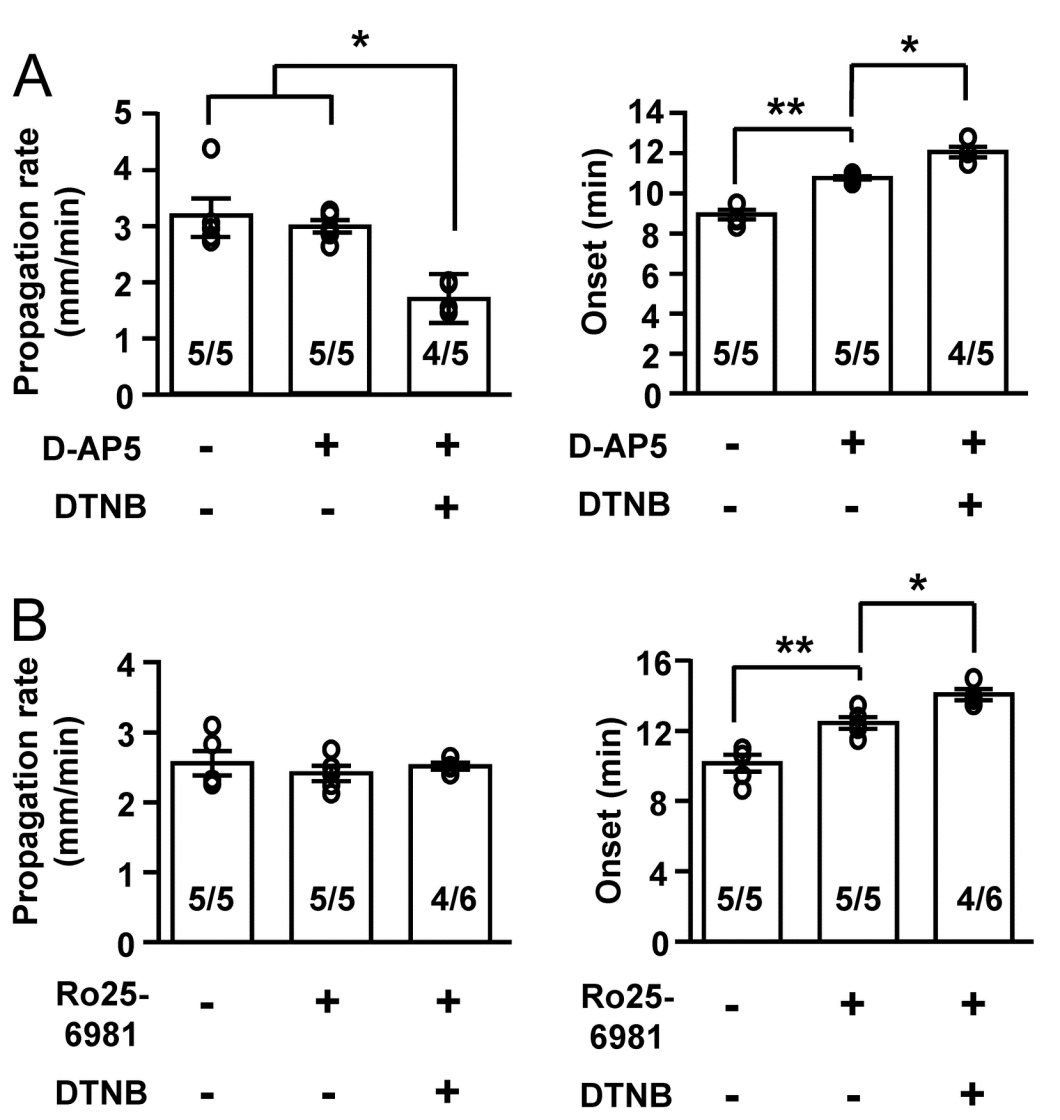
DTNB enhanced effects of synthetic NMDA receptor antagonists on OGD-SD. **A**: When tested on OGD-SD, the competitive NMDAR antagonist D-AP5 (25 µM) was without effect on propagation rate, but caused a significant delay in SD onset. DTNB significantly enhanced the effects of D-AP5, so that propagation rate was decreased and onset time was further delayed. In one experiment with D-AP5 and DTNB, SD could not be detected. **B**: The allosteric inhibitor Ro25-6981 (10 µM) significantly delayed OGD-SD onset and the inhibitory effect was larger in the DTNB treated slices. Unlike D-AP5, Ro25-6981 was without effect on SD propagation in control and DTNSB treated slices. In two experiments with Ro25-6981 and DTNB, SD was not detected and statistical tests were conducted by using values obtained from successfully generated SDs. Numbers in the bars indicate the incidence of SD. These experiments were conducted in slices obtained from male C57BL/6 animals. * p<0.05, ** p<0.01 .

In a final set of experiments, we examined whether the Zn^2+^ sensitivity of OGD-SD were matched by effects of synthetic NMDAR antagonists (Figure **6**). Figure 6A shows that the concentration of AP5 tested (25 µM) had no effect on OGD-SD propagation, but delayed onset of the event. Consistent with the effects on Zn^2+^ described above, when tested in DTNB, AP5 inhibited SD propagation, and effects on onset were further enhanced. An allosteric NMDAR inhibitor (Ro25-6985) was tested at a concentration that, like AP5 was without effect on propagation rate and significantly delayed OGD-SD onset (Figure 6B). Similar to AP5, DTNB further delayed OGD-SD onset, but Ro25-6981 was without effect on the propagation rate of SD in both control and DTNB treated slices, possibly due to its subunit selectivity or its mechanism of inhibition (see Discussion). These results suggest that anoxic redox modulation can significantly affect the efficacies of some NMDAR antagonists on OGD-SD. 

## Discussion

### 1. General

We previously showed that SDs generated by localized KCl applications were inhibited by extracellular Zn^2+^ [7], and the present study extends these findings to other forms of SD that may be more relevant for ischemic injury. Extracellular Zn^2+^ inhibited normoxic SDs (K-SD and ouabain-SDs generated in 95% and/or 21% O_2_), but did not inhibit anoxic SDs (OGD-SD, ouabain-SD in 0% O_2_). These effects of hypoxia may be contributed to by oxygen-dependent redox potential shifts, which could result in extracellular modification of Zn^2+^ targets such as NMDARs. These findings were also extended to inhibitory effects of synthetic NMDAR antagonists. Together, these results provide an additional mechanism for the lack of effectiveness of pharmacological inhibitors for some forms of SD, which could be important for design of therapeutic interventions.

### 2. Implications for Zn^2+^ in stroke

The oxygen-dependence of the sensitivity of SD to Zn^2+^ adds complexity to the effects of Zn^2+^ in ischemic brain injury. Following ischemia, extracellular Zn^2+^ concentrations elevate [29], and this could be contributed to by spontaneous repetitive SDs, which are frequent in the post-ischemic period [30] and which release significant amounts of Zn^2+^ from presynaptic vesicles into the extracellular space [6]. Recent studies have demonstrated that SD generated following focal stroke in rodents can circularly propagate around the infarct core [31]. The present results suggest that extracellular Zn^2+^ increases may help to confine SD propagation to metabolically compromised vulnerable tissues, by selectively inhibiting propagation of SDs into surrounding well-oxygenated tissues. The same considerations may also apply in more complex clinical strokes, involving occlusion of small arteries that create complex oxygen gradients around an ischemic core where SDs may initiate. 

As noted in the Introduction, the initiation of SD can be facilitated by intracellular accumulation of Zn^2+^. Thus intracellular and extracellular Zn^2+^ effects appear to oppose each other for the generation of SD. Intracellular Zn^2+^ was suggested to facilitate SD initiation by inhibiting energy metabolism [15], and a significant contribution of intracellular Zn^2+^ accumulation was observed for ouabain and OGD-SD, but not for K-SD [14]. The net effects of Zn^2+^ likely depend on the levels of Zn^2+^ achieved in the two compartments, the sensitivity of the SD stimulus to intracellular accumulation, and as discussed here, whether hypoxia blunts the effectiveness of extracellular inhibition. Consistent with this hypothesis, the present study showed that ZnCl_2_ exposures increased the threshold for K-SD, but were without effects on ouabain-SD or OGD-SD (Figure 3). Furthermore, some inhibitory effect on OGD-SD could be revealed, if DTNB were used to increase the effectiveness of extracellular Zn^2+^ (Figure 4). These observations suggest that in metabolically compromised brain regions following stroke, the net effect of Zn^2+^ release and accumulation is likely to facilitate SD onset. Such effects are expected to combine with a number of other deleterious consequences of intracellular Zn^2+^ accumulation to contribute to neuronal injury [9]. 

### 3. Redox modulation of Zn^2+^ sensitivity

The present study suggests that extracellular redox potential shifts (toward reduction) in hypoxia resulted in decreased sensitivity of SD to extracellular Zn^2+^. Thus exogenous application of oxidants (DTNB, GSSG) was sufficient to render hypoxic SDs sensitive to Zn^2+^. It is technically challenging to test redox potential shifts and Zn^2+^ sensitivity in the reverse experiment, i.e. whether an exogenous reducing agent can make Zn^2+^ less effective. This is because most reducing reagents also chelate Zn^2+^ [8]. While we have observed that the reducing reagent DTT abolished the Zn^2+^ sensitivity of K-SD (data not shown), it is quite possible that this effect of DTT is simply due to reduced extracellular Zn^2+^ concentrations. It is also noted that metallothionein or other Zn^2+^-binding thiols may be released from neurons [32], and the Zn^2+^-binding capacity of these proteins could be decreased by hypoxia. It is conceivable that hypoxic regulation of such a mechanism could make some contribution to the effects described here. However, it is not yet known whether Zn^2+^ binding protein release is significant following SD, and the fact that a similar sensitivity to hypoxia was also observed with other NMDA antagonists makes it unlikely that Zn^2+^-binding protein regulation is a major contributor to the effects seen here.

As described in the Results section, a previous study has clearly shown a strong effect of redox modulation on SD. DTNB (2 mM) postponed the onset of SD generated by hypoxia, and the effect was concluded to be mediated by redox activation of BK channels [23]. We observed a similar inhibition of OGD-SD by 2 mM DTNB, but found that a lower concentration (0.5 mM) could be used to minimize the inhibitory effect on OGD-SD by DTNB itself. Such a concentration-dependent effect of DTNB could be due to the low membrane permeability of DTNB, as intracellular sites of BK channels were shown to be responsible for potentiation by oxidation [33,34]. Thus it is possible that DTNB at a higher concentration (2 mM) could modulate intracellular sites (e.g. BK channels) and prevent SD whereas, at a lower concentration (0.5 mM), DTNB preferentially modulated extracellular sites and revealed sensitivity to extracellular Zn^2+^. 

### 4. Hypoxic modulation of NMDARs

As discussed previously [7], inhibition of NMDARs is one possible target that could explain the inhibitory effects of extracellular Zn^2+^ on SD. The present observations that both NMDAR currents and SD were similarly sensitive to hypoxia-dependent redox modulation are also consistent with this hypothesis. 

The hypoxic regulation of Zn^2+^ sensitivity of NMDARs (Figure 5) could be anticipated from previous studies. Oxygen-sensitive sites have been reported in NMDARs, and localized to the N-terminal region [35]. These sites are located close to Zn^2+^ binding sites [26]. In addition, redox modulation was shown to modify both the Zn^2+^ sensitivity of NMDAR currents in cultured cortical neurons [27] and the protective effects of synthetic NMDAR antagonists in hippocampal slice cultures [28]. The effect of redox modulation on NMDAR currents seen in the present study with hypoxia was somewhat larger than that demonstrated with chemical modification [27]. This could be explained by differences in methods used for redox modulation, different GluN2 subunit composition between brain tissues from adults vs. embryos, or possibly additional effects of hypoxia such as changes in extracellular pH that could modify Zn^2+^ sensitivity NMDAR [36].

The present study used OGD conditions that allowed assessment of changes in NMDAR sensitivity. Thus a small amount of glucose (1.5mM) was maintained in the OGD challenges, and under these conditions SD was reliably generated, and effects of Zn^2+^ and synthetic NMDAR antagonists could be effectively modulated by changes in O_2_ availability or redox modulators. It is emphasized that complete removal of both oxygen and glucose can render OGD-SD in brain slices completely resistant to NMDAR antagonists [37,38]. The fact that small changes in metabolic substrate availability can strongly influence NMDAR sensitivity seems consistent with results from experimental stroke studies. Thus complete NMDAR antagonist resistance has been shown in SD observed during global ischemia [39-41], whereas significant antagonist sensitivity has been demonstrated in SD generated following focal ischemia [30,42-44]. The mechanisms for reduced effectiveness of NMDAR antagonists in ischemia have been studied *in vivo* and in brain slices, and attributed to elevated baseline potassium concentrations [45] that occur in metabolically compromised brain tissue [46]. The redox modification studied here could be an additional mechanism that limits the inhibitory effects of NMDAR antagonists on SD in hypoxic tissues. It is also possible that redox modulation of NMDARs may affect Zn^2+^ sensitivity in a range of other pathophysiological conditions. For example, a shift from oxidized to reduced NMDARs can contribute to seizure-like activity [25], and it will be of interest to determine whether oxidizing agents significantly increase the effectiveness of Zn^2+^ block in epileptogenic conditions, or related disorders that involve increased excitability and/or local tissue hypoxia. 

## References

[1] Somjen GG (2001) Mechanisms of spreading depression and hypoxic spreading depression-like depolarization. Physiol Rev 81: 1065-1096. PubMed: 11427692. 11427692 10.1152/physrev.2001.81.3.1065

[2] Hartings JA, Bullock MR, Okonkwo DO, Murray LS, Murray GD et al. (2011) Spreading depolarisations and outcome after traumatic brain injury: a prospective observational study. Lancet Neurol 10: 1058-1064. doi:10.1016/S1474-4422(11)70243-5. PubMed: 22056157. 22056157

[3] Dohmen C, Sakowitz OW, Fabricius M, Bosche B, Reithmeier T et al. (2008) Spreading depolarizations occur in human ischemic stroke with high incidence. Ann Neurol 63: 720-728. doi:10.1002/ana.21390. PubMed: 18496842. 18496842

[4] Lauritzen M, Dreier JP, Fabricius M, Hartings JA, Graf R et al. (2011) Clinical relevance of cortical spreading depression in neurological disorders: migraine, malignant stroke, subarachnoid and intracranial hemorrhage, and traumatic brain injury. J Cereb Blood Flow Metab 31: 17-35. doi:10.1038/jcbfm.2010.191. PubMed: 21045864. 21045864 PMC3049472

[5] Dreier JP (2011) The role of spreading depression, spreading depolarization and spreading ischemia in neurological disease. Nat Med 17: 439-447. doi:10.1038/nm.2333. PubMed: 21475241. 21475241

[6] Carter RE, Aiba I, Dietz RM, Sheline CT, Shuttleworth CW (2011) Spreading depression and related events are significant sources of neuronal Zn2+ release and accumulation. J Cereb Blood Flow Metab 31: 1073-1084. doi:10.1038/jcbfm.2010.183. PubMed: 20978516. 20978516 PMC3070966

[7] Aiba I, Carlson AP, Sheline CT, Shuttleworth CW (2012) Synaptic release and extracellular actions of Zn2+ limit propagation of spreading depression and related events in vitro and in vivo. J Neurophysiol 107: 1032-1041. doi:10.1152/jn.00453.2011. PubMed: 22131381. 22131381 PMC3289481

[8] Paoletti P, Ascher P, Neyton J (1997) High-affinity zinc inhibition of NMDA NR1-NR2A receptors. J Neurosci 17: 5711-5725. PubMed: 9221770. 9221770 10.1523/JNEUROSCI.17-15-05711.1997 PMC6573217

[9] Shuttleworth CW, Weiss JH (2011) Zinc: new clues to diverse roles in brain ischemia. Trends Pharmacol Sci 32: 480-486. doi:10.1016/j.tips.2011.04.001. PubMed: 21621864. 21621864 PMC3148334

[10] Sensi SL, Paoletti P, Bush AI, Sekler I (2009) Zinc in the physiology and pathology of the CNS. Nat Rev Neurosci 10: 780-791. doi:10.1038/nrn2734. PubMed: 19826435. 19826435

[11] Qian J, Xu K, Yoo J, Chen TT, Andrews G et al. (2011) Knockout of Zn transporters Zip-1 and Zip-3 attenuates seizure-induced CA1 neurodegeneration. J Neurosci 31: 97-104. doi:10.1523/JNEUROSCI.5162-10.2011. PubMed: 21209194. 21209194 PMC3078714

[12] Koh JY, Suh SW, Gwag BJ, He YY, Hsu CY et al. (1996) The role of zinc in selective neuronal death after transient global cerebral ischemia. Science 272: 1013-1016. doi:10.1126/science.272.5264.1013. PubMed: 8638123. 8638123

[13] Calderone A, Jover T, Mashiko T, Noh KM, Tanaka H et al. (2004) Late calcium EDTA rescues hippocampal CA1 neurons from global ischemia-induced death. J Neurosci 24: 9903-9913. doi:10.1523/JNEUROSCI.1713-04.2004. PubMed: 15525775. 15525775 PMC6730239

[14] Dietz RM, Weiss JH, Shuttleworth CW (2008) Zn2+ influx is critical for some forms of spreading depression in brain slices. J Neurosci 28: 8014-8024. doi:10.1523/JNEUROSCI.0765-08.2008. PubMed: 18685026. 18685026 PMC2577031

[15] Dietz RM, Weiss JH, Shuttleworth CW (2009) Contributions of Ca2+ and Zn2+ to spreading depression-like events and neuronal injury. J Neurochem 109 Suppl 1: 145-152. doi:10.1111/j.1471-4159.2009.05853.x. PubMed: 19393021. 19393021 PMC2692040

[16] Aiba I, Shuttleworth CW (2012) Sustained NMDAR activation by spreading depolarizations can initiate excitotoxic injury in metabolically compromised neurons. J Physiol. 10.1113/jphysiol.2012.234476 PMC3528997 22907056

[17] Rumschik SM, Nydegger I, Zhao J, Kay AR (2009) The interplay between inorganic phosphate and amino acids determines zinc solubility in brain slices. J Neurochem 108: 1300-1308. doi:10.1111/j.1471-4159.2009.05880.x. PubMed: 19183267. 19183267 PMC2720156

[18] Gribkoff VK, Bauman LA (1992) Endogenous adenosine contributes to hypoxic synaptic depression in hippocampus from young and aged rats. J Neurophysiol 68: 620-628. PubMed: 1527579. 1527579 10.1152/jn.1992.68.2.620

[19] Lindquist BE, Shuttleworth CW (2012) Adenosine receptor activation is responsible for prolonged depression of synaptic transmission after spreading depolarization in brain slices. Neuroscience 223: 365-376. doi:10.1016/j.neuroscience.2012.07.053. PubMed: 22864185. 22864185 PMC3489063

[20] Wagner CA, Waldegger S, Osswald H, Biber J, Murer H et al. (1996) Heavy metals inhibit Pi-induced currents through human brush-border NaPi-3 cotransporter in Xenopus oocytes. Am J Physiol 271: F926-F930. PubMed: 8898024. 8898024 10.1152/ajprenal.1996.271.4.F926

[21] Choi Y, Chen HV, Lipton SA (2001) Three pairs of cysteine residues mediate both redox and zn2+ modulation of the nmda receptor. J Neurosci 21: 392-400. PubMed: 11160420. 11160420 10.1523/JNEUROSCI.21-02-00392.2001 PMC6763802

[22] Herin GA, Du S, Aizenman E (2001) The neuroprotective agent ebselen modifies NMDA receptor function via the redox modulatory site. J Neurochem 78: 1307-1314. doi:10.1046/j.1471-4159.2001.00517.x. PubMed: 11579139. 11579139

[23] Hepp S, Gerich FJ, Müller M (2005) Sulfhydryl oxidation reduces hippocampal susceptibility to hypoxia-induced spreading depression by activating BK channels. J Neurophysiol 94: 1091-1103. doi:10.1152/jn.00291.2005. PubMed: 15872065. 15872065

[24] Gozlan H, Diabira D, Chinestra P, Ben-Ari Y (1994) Anoxic LTP is mediated by the redox modulatory site of the NMDA receptor. J Neurophysiol 72: 3017-3022. PubMed: 7897507. 7897507 10.1152/jn.1994.72.6.3017

[25] Sanchez RM, Wang C, Gardner G, Orlando L, Tauck DL et al. (2000) Novel role for the NMDA receptor redox modulatory site in the pathophysiology of seizures. J Neurosci 20: 2409-2417. PubMed: 10704515. 10704515 10.1523/JNEUROSCI.20-06-02409.2000 PMC6772486

[26] Paoletti P, Neyton J (2007) NMDA receptor subunits: function and pharmacology. Curr Opin Pharmacol 7: 39-47. doi:10.1016/j.coph.2006.08.011. PubMed: 17088105. 17088105

[27] Tang LH, Aizenman E (1993) The modulation of N-methyl-D-aspartate receptors by redox and alkylating reagents in rat cortical neurones in vitro. J Physiol 465: 303-323. PubMed: 7693919. 7693919 10.1113/jphysiol.1993.sp019678 PMC1175431

[28] Pringle AK, Self J, Eshak M, Iannotti F (2000) Reducing conditions significantly attenuate the neuroprotective efficacy of competitive, but not other NMDA receptor antagonists in vitro. Eur J Neurosci 12: 3833-3842. doi:10.1046/j.1460-9568.2000.00272.x. PubMed: 11069578. 11069578

[29] Kitamura Y, Iida Y, Abe J, Mifune M, Kasuya F et al. (2006) Release of vesicular Zn2+ in a rat transient middle cerebral artery occlusion model. Brain. Res Bull 69: 622-625. doi:10.1016/j.brainresbull.2006.03.004. 16716828

[30] Hartings JA, Rolli ML, Lu XC, Tortella FC (2003) Delayed secondary phase of peri-infarct depolarizations after focal cerebral ischemia: relation to infarct growth and neuroprotection. J Neurosci 23: 11602-11610. PubMed: 14684862. 14684862 10.1523/JNEUROSCI.23-37-11602.2003 PMC6740954

[31] Nakamura H, Strong AJ, Dohmen C, Sakowitz OW, Vollmar S et al. (2010) Spreading depolarizations cycle around and enlarge focal ischaemic brain lesions. Brain 133: 1994-2006. doi:10.1093/brain/awq117. PubMed: 20504874. 20504874 PMC2892938

[32] Chung RS, Penkowa M, Dittmann J, King CE, Bartlett C et al. (2008) Redefining the role of metallothionein within the injured brain: extracellular metallothioneins play an important role in the astrocyte-neuron response to injury. J Biol Chem 283: 15349-15358. doi:10.1074/jbc.M708446200. PubMed: 18334482. 18334482 PMC3258880

[33] Gong L, Gao TM, Huang H, Tong Z (2000) Redox modulation of large conductance calcium-activated potassium channels in CA1 pyramidal neurons from adult rat hippocampus. Neurosci Lett 286: 191-194. doi:10.1016/S0304-3940(00)01121-6. PubMed: 10832017. 10832017

[34] Park MK, Bae YM, Lee SH, Ho WK, Earm YE (1997) Modulation of voltage-dependent K+ channel by redox potential in pulmonary and ear arterial smooth muscle cells of the rabbit. Pflugers Arch 434: 764-771. doi:10.1007/s004240050463. PubMed: 9306010. 9306010

[35] Takahashi H, Shin Y, Cho SJ, Zago WM, Nakamura T et al. (2007) Hypoxia enhances S-nitrosylation-mediated NMDA receptor inhibition via a thiol oxygen sensor motif. Neuron 53: 53-64. doi:10.1016/j.neuron.2006.11.023. PubMed: 17196530. 17196530 PMC1855274

[36] Low CM, Zheng F, Lyuboslavsky P, Traynelis SF (2000) Molecular determinants of coordinated proton and zinc inhibition of N-methyl-D-aspartate NR1/NR2A receptors. Proc Natl Acad Sci U S A 97: 11062-11067. doi:10.1073/pnas.180307497. PubMed: 10984504. 10984504 PMC27148

[37] Jarvis CR, Anderson TR, Andrew RD (2001) Anoxic depolarization mediates acute damage independent of glutamate in neocortical brain slices. Cereb Cortex 11: 249-259. doi:10.1093/cercor/11.3.249. PubMed: 11230096. 11230096

[38] Joshi I, Andrew RD (2001) Imaging anoxic depolarization during ischemia-like conditions in the mouse hemi-brain slice. J Neurophysiol 85: 414-424. PubMed: 11152742. 11152742 10.1152/jn.2001.85.1.414

[39] Murphy TH, Li P, Betts K, Liu R (2008) Two-photon imaging of stroke onset in vivo reveals that NMDA-receptor independent ischemic depolarization is the major cause of rapid reversible damage to dendrites and spines. J Neurosci 28: 1756-1772. doi:10.1523/JNEUROSCI.5128-07.2008. PubMed: 18272696. 18272696 PMC6671530

[40] Hernándéz-Cáceres J, Macias-González R, Brozek G, Bures J (1987) Systemic ketamine blocks cortical spreading depression but does not delay the onset of terminal anoxic depolarization in rats. Brain Res 437: 360-364. doi:10.1016/0006-8993(87)91652-0. PubMed: 3435842. 3435842

[41] Lauritzen M, Hansen AJ (1992) The effect of glutamate receptor blockade on anoxic depolarization and cortical spreading depression. J Cereb Blood Flow Metab 12: 223-229. doi:10.1038/jcbfm.1992.32. PubMed: 1312539. 1312539

[42] Gill R, Andiné P, Hillered L, Persson L, Hagberg H (1992) The effect of MK-801 on cortical spreading depression in the penumbral zone following focal ischaemia in the rat. J Cereb Blood Flow Metab 12: 371-379. doi:10.1038/jcbfm.1992.54. PubMed: 1314840. 1314840

[43] Ohta K, Graf R, Rosner G, Heiss WD (2001) Calcium ion transients in peri-infarct depolarizations may deteriorate ion homeostasis and expand infarction in focal cerebral ischemia in cats. Stroke 32: 535-543. doi:10.1161/01.STR.32.2.535. PubMed: 11157194. 11157194

[44] Iijima T, Mies G, Hossmann KA (1992) Repeated negative DC deflections in rat cortex following middle cerebral artery occlusion are abolished by MK-801: effect on volume of ischemic injury. J Cereb Blood Flow Metab 12: 727-733. doi:10.1038/jcbfm.1992.103. PubMed: 1506440. 1506440

[45] Petzold GC, Windmüller O, Haack S, Major S, Buchheim K et al. (2005) Increased extracellular K+ concentration reduces the efficacy of N-methyl-D-aspartate receptor antagonists to block spreading depression-like depolarizations and spreading ischemia. Stroke 36: 1270-1277. doi:10.1161/01.STR.0000166023.51307.e0. PubMed: 15879337. 15879337

[46] Nedergaard M, Hansen AJ (1993) Characterization of cortical depolarizations evoked in focal cerebral ischemia. J Cereb Blood Flow Metab 13: 568-574. doi:10.1038/jcbfm.1993.74. PubMed: 8314912. 8314912

